# Long-term impacts of disturbance on nitrogen-cycling bacteria in a New England salt marsh

**DOI:** 10.3389/fmicb.2015.00046

**Published:** 2015-02-04

**Authors:** Anne E. Bernhard, Courtney Dwyer, Adrian Idrizi, Geoffrey Bender, Rachel Zwick

**Affiliations:** Biology Department, Connecticut College, New London, CTUSA

**Keywords:** *amo*A, disturbance, *nir*S, restoration, salt marsh

## Abstract

Recent studies on the impacts of disturbance on microbial communities indicate communities show differential responses to disturbance, yet our understanding of how different microbial communities may respond to and recover from disturbance is still rudimentary. We investigated impacts of tidal restriction followed by tidal restoration on abundance and diversity of denitrifying bacteria, ammonia-oxidizing bacteria (AOB), and ammonia-oxidizing archaea (AOA) in New England salt marshes by analyzing *nir*S and bacterial and archaeal *amo*A genes, respectively. TRFLP analysis of *nir*S and betaproteobacterial *amo*A genes revealed significant differences between restored and undisturbed marshes, with the greatest differences detected in deeper sediments. Additionally, community patterns indicated a potential recovery trajectory for denitrifiers. Analysis of archaeal *amo*A genes, however, revealed no differences in community composition between restored and undisturbed marshes, but we detected significantly higher gene abundance in deeper sediment at restored sites. Abundances of *nir*S and betaproteobacterial *amo*A genes were also significantly greater in deeper sediments at restored sites. Porewater ammonium was significantly higher at depth in restored sediments compared to undisturbed sediments, suggesting a possible mechanism driving some of the community differences. Our results suggest that impacts of disturbance on denitrifying and ammonia-oxidizing communities remain nearly 30 years after restoration, potentially impacting nitrogen-cycling processes in the marsh. We also present data suggesting that sampling deeper in sediments may be critical for detecting disturbance effects in coastal sediments.

## INTRODUCTION

Nitrification, the oxidation of ammonia to nitrate, and denitrification, the reduction of oxidized nitrogen to dinitrogen gas, play critical roles in determining the availability of nitrogen in salt marshes. Nitrogen is arguably one of the most important nutrients regulating the high productivity reported in many salt marshes (Vitousek et al., 2002), and its availability is controlled primarily by the activity of microorganisms. Additionally, nitrification and denitrification are often coupled (Risgaard-Petersen, 2003), and limited evidence from studies of nitrifiers and denitrifiers suggests that process rates are linked to diversity and community composition of the microbes responsible (Cavigelli and Robertson, 2000; Horz et al., 2004; Webster et al., 2005; Bernhard et al., 2007). Despite their importance to ecosystem productivity, our understanding of what regulates nitrifying and denitrifying communities, how they interact with each other, and how they may respond to ecosystem perturbations remains relatively enigmatic.

New England salt marshes have undergone extensive disturbances in the last century, including nutrient loading, ditching to control mosquitoes, and other modifications to normal tidal flow for recreational and commercial purposes (Gedan et al., 2009). In the last few decades, as we have realized the important roles that salt marshes play in maintaining healthy coastlines, extensive efforts have been underway to restore these vital habitats to their original state. Much of the research on the recovery of salt marshes has focused on changes in macrobiota and biogeochemical processes (see reviews by Warren et al., 2002; Callaway, 2005), with potential impacts on the microbiota only recently coming under scrutiny.

Restricting access of tidal waters to marshes, commonly by diking or impounding, obviously reduces salinity, but also leads to other changes in hydrology and biogeochemical processes important to salt marsh ecology. As the marsh freshens, the water table is lowered, resulting in higher oxygen levels and increased aerobic decomposition, leading to sediment subsidence (Portnoy, 1999). Reduction in salinity also means a reduction in the supply of sulfate, leading to a switch from sulfate reduction to methanogenesis as the primary pathway of anaerobic decomposition (van Proosdij et al., 2010). Because methanogenesis is less energetically favorable, decomposition decreases, as does mineralization of N and P, leaving sediments with higher organic matter. Consequently, sediment chemistry has been shown to differ significantly in impounded marshes compared to unrestricted ones (Portnoy, 1999). When saltwater is restored to the marsh, sulfides increase and oxygen decreases. Depending on the hydrology of the marsh, sediments may also experience changes in pH and availability of inorganic nutrients (Portnoy and Giblin, 1997). Changes in grain size and porosity of sediment due to altered sedimentation patterns may also impact retention of N (Zedler and Kercher, 2005). Changes in sediment chemistry are expected to have significant impacts on the sediment microbial communities that drive much of the biogeochemical processes.

To date, a handful of studies have investigated the impacts of disturbance on salt marsh microbes and, surprisingly, have yielded few significant impacts. In response to nutrient manipulations, minimal impacts on bacterial communities have been reported (Lovell et al., 2001; Bowen et al., 2009). In another study, Bowen et al. (2011) showed no significant differences in the total bacterial communities or the denitrifying communities in two marshes after acute and chronic fertilization. In a study of nitrifying microbes in one of the same marshes sampled by Bowen et al. (2011), significant impacts of fertilization were detected on ammonia oxidizing bacteria, but not on archaea (Peng et al., 2013). And, Bernhard et al. (2012) reported no differences in microbial community composition, but greater variability, in impounded and subsequently restored marshes compared to undisturbed marshes. The minimal impacts of disturbance on salt marsh microbial communities are somewhat surprising given that others have reported significant impacts in other habitats (Downing and Leibold, 2010; Shade et al., 2011; Berga et al., 2012), and that in some cases, significant differences were reported for biogeochemical processes in the marsh (e.g., Hamersley and Howes, 2005). The limited data from salt marsh studies suggest that microbial communities may exhibit different levels of resilience or resistance (as defined in Allison and Martiny, 2008) to perturbations or that we have not sampled adequately, and that our understanding of how disturbance theory applies to microbial communities is still rudimentary (see Shade et al., 2012).

In this study, we investigated the long-term impacts of impoundment (tidal restriction) and subsequent tidal flow restoration (considered press, or chronic, disturbances) on ammonia-oxidizing bacteria (AOB), ammonia-oxidizing archaea (AOA), and denitrifying bacteria in Connecticut salt marshes. We chose nitrifiers and denitrifiers as the targets partly because of differential responses to fertilization these groups have shown in previous studies (Bowen et al., 2011; Peng et al., 2013). Furthermore, a recent study in the same Connecticut marshes (Bernhard et al., 2012) showed greater spatial and temporal variability of bacterial communities in restored marshes, but whether such variability might impact ecosystem services, such as nutrient cycling, in the marshes remains uncertain. Our goals in this study were to determine if the disturbances to the marshes (impoundment and subsequent tidal flow restoration) have left lasting effects on microbial communities involved in two critical nitrogen-cycling processes, and if the response patterns of the functional groups are similar, suggesting a broader-scale impact to nitrogen-cycling in the marsh.

## MATERIALS AND METHODS

### SITE DESCRIPTION

Samples were collected from the Wequetequock-Pawcatuck (known as Barn Island) and Cottrell salt marshes in southeastern Connecticut. Complete site descriptions and the full management history of the sites have been previously described (Warren and Neiring, 1993; Bernhard et al., 2012). Briefly, four marshes within the Barn Island system were impounded (hereafter referred to as Impoundments 1–4) in the late 1940s. Starting in 1978, tidal flow was restored to the four marshes over a period of 13 years, with Impoundments 1 and 2 restored first, followed by Impoundment 4 in 1987, and finally, Impoundment 3 in 1991. Two undisturbed marshes in Barn Island (Wequetequock Cove and Headquarters) and two additional sites in the nearby Cottrell marsh were selected as reference marshes for comparison. Dominant vegetation at all sites was *Spartina patens*.

### SAMPLE COLLECTION

Triplicate sediment cores (6.5 cm diameter) were collected from each of the eight sites in July 2006 and sectioned into 0–2 cm and 6–8 cm horizons. Each horizon was homogenized and aliquoted for DNA extraction, porewater analyses (salinity, pH, % water, and NH_4_^+^), and dry wt determination. Methods for DNA extraction, porewater analyses, and dry wt determination have been previously published (Nelson et al., 2009; Bernhard et al., 2012).

### QUANTITATIVE PCR

*nir*S genes were quantified by real-time PCR using the primers *nir*S-1F and *nir*S-3R (Braker et al., 1998). All 20 μl reactions were run in an iCycler (BioRad) with ca. 5–10 ng DNA, SYBR Green I super mix (BioRad), 0.5 μM of each primer, 0.008% bovine serum albumin using the following conditions for 40 cycles: 95^∘^C for 15 s, 54^∘^C for 20 s, 72^∘^C for 30 s. To monitor product specificity, we conducted melt curve analysis (95^∘^C for 1 min, 54^∘^C for 1 min, and then 0.5^∘^C increase every 10 s, with fluorescence read continuously) after each run. Gene abundances were estimated by comparison to known concentrations of a plasmid containing a cloned *nir*S gene. Concentrations of the plasmid ranged from 1 pg to 1 fg. Bacterial 16S rRNA genes and *amo*A genes were quantified as previously described (Moin et al., 2009; Bernhard et al., 2012; Peng et al., 2013). PCR efficiencies were 94.3 ± 0.14% (*nir*S), 93.4 ± 4.7% (AOB), 99.6 ± 10.0% (AOA), and 89.2% (bacterial 16S rRNA).

### COMMUNITY FINGERPRINTS

The *nir*S gene was amplified from all samples in triplicate using the primers *nir*S-1F and *nir*S-6R (Braker et al., 1998). The *nir*S-1F primer was labeled at the 5^′^-end with 6-FAM. PCRs were run with the following cycle conditions: 30 cycles of 95^∘^C for 15 s, 54^∘^C for 30 s, and 72^∘^C for 90 s, followed by a final elongation of 5 min at 72^∘^C. PCR products were confirmed by comparison to a DNA molecular weight ladder by electrophoresis analysis in a 1% agarose gel. Positive PCR products were then digested with 10 units of *Hha*I overnight at 37^∘^C followed by by ethanol precipitation. The restriction endonuclease *Hha*I was found to provide the greatest discrimination based on *in silico* analysis of *nir*S sequences (data not shown). An additional advantage of using *Hha*I, is that it produces a 3^′^ overhang, and therefore only yields the original terminal restriction fragment (TRF) without producing artifacts due to residual polymerase activity (Hartman et al., 2007).

Betaproteobacterial and archaeal *amo*A genes were processed for Terminal restriction fragment length polymorphism (TRFLP) analysis as previously described (Bernhard et al., 2005; Peng et al., 2013). Digests of *amo*A and *nir*S genes were resuspended in 5 μl of deionized H_2_O, 0.2 μl of the internal size standard, GS500-ROX (Applied Biosystems Inc., Fremont, CA, USA), and 10 μl of Hi-Di Formamide (ABI) and sent to the Biotechnology Resource Center at Cornell University () for analysis on an Applied BioSystems 3730xl DNA Analyzer.

Terminal restriction fragment sizes and relative abundances for both *amo*A and *nir*S genes were estimated using GeneMarker software, version 1.4 (SoftGenetics, State College, PA, USA). Since betaprotebacterial and archaeal *amo*A diversity in New England salt marshes has been relatively well-characterized (Bernhard et al., 2005; Moin et al., 2009; Peng et al., 2013), we included only TRFs previously identified from published *amo*A sequences in our analysis to minimize the impact of TRFLP artifacts. However, the *nir*S genes from New England salt marshes have not been as well characterized as the *amo*A genes, so we analyzed two different data sets for *nir*S. The first data set included all detectable TRFs in the analysis, but likely included artifacts due to chimera or heteroduplex formation. The second data set included only the TRFs that were confirmed by sequence analysis from our samples or from publically available sequences. Because the results were similar between the two data sets based on multivariate analyses (data not shown), we present the results from the second data set that included only TRFs that were represented by a *nir*S sequence. We acknowledge that this data set may not include all *nir*S TRFs since the *nir*S sequence database for salt marshes is likely incomplete, but we chose to focus on the more conservative approach to avoid including potential artifacts.

### STATISTICAL ANALYSES

Terminal restriction fragment length polymorphism profiles were compared using PC-Ord version 6 (McCune and Mefford, 1999). The relative abundance data were transformed by an arcsine square root function to reduce skew. Non-metric multidimensional scaling (NMS; Kruskal, 1964) was used to ordinate samples in gene fragment space, using the SØrenson’s distance measure. The autopilot option was set to the slow and thorough level for all ordinations. Monte Carlo tests were run to confirm that results obtained were significantly better than would be obtained from randomized data. Additionally, the proportion of variance explained by each axis and the cumulative variance explained was determined by calculating the coefficient of determination between distances in ordination space and distances in the original *p*-dimensional space. Correlation coefficients in the ordination space were determined for environmental variables and TRFs by rotating the ordination to maximize the coefficient on one axis (Varimax rotation) in order to facilitate detecting clusters of samples (McCune and Grace, 2002).

Multi-response permutation procedure (MRPP), a non-parametric test, was used to test for differences between restored and undisturbed sites and among different sites. MRPP is a variant of analysis of similarity and provides a measure of the effect and *p*-value when testing for differences between two or more groups defined by the user (McCune and Grace, 2002).

Differences in relative abundance of TRFs and abundance of genes between restored and undisturbed marshes were detected by Student’s *t*-tests on arcsin square root transformed data using InStat 3.0b (GraphPad Software, Inc.). In some cases, data transformation was not sufficient to meet the assumptions of the *t*-test, so we applied the nonparametric Mann–Whitney test. Statistical significance for all analyses was set at α = 0.05.

### SEQUENCE ANALYSIS OF *nir*S GENES

Since betaproteobacterial and archaeal *amo*A genes have been previously characterized in the marshes studied here (Moin et al., 2009), we focused our sequencing efforts only on the *nir*S genes. Our intention was not to fully describe the phylogeny of *nir*S genes, but rather to identify TRFs of the most frequently detected *nir*S populations to include in community analysis.

The *nir*S gene was amplified from DNA from 0 to 2 cm and 6 to 8 cm horizons from cores collected from Wequetequock Cove and Impoundments 1 and 4 using the primers *nir*S1F and *nir*S6R (Braker et al., 1998). PCR products were cloned into the pSC vector using the StrataClone PCR Cloning kit (Stratagene, Agilient Technologies, Santa Clara, CA, USA) following the manufacturer’s recommendations. Thirty-three clones from each of the six libraries were screened with the vector-specific primers M13F and M13R. Clones containing the correct size insert were sequenced by High Throughput Sequencing Solutions (University of Washington, Department of Genome Sciences, Seattle, WA, USA) using the vector-specific primers T3 and T7. Nucleotide sequences for *nir*S have been deposited in Genbank under the accession numbers KF895915-KF896071.

## RESULTS

### POREWATER ANALYSIS

Analysis of porewater chemistry revealed some significant differences in conditions between restored and undisturbed sites and between surface and deep sediments (**Table 1**). Both pH and ammonium were significantly higher in restored sites compared to undisturbed sites, but only in the deeper sediment for ammonium and surface sediments for pH (pH data for deep sediments was not available). We also detected significant differences in salinity between surface and deeper sediment, but only at the restored sites. We did not measure nitrate concentrations from the samples in this study, but previous measurements from other sampling dates at the same sites do not indicate significant differences in nitrate concentrations between restored and undisturbed sites (Bernhard, unpublished).

**Table 1:** Mean (±SE) salinity, pH, ammonium, and % water in porewater of sediments from 0 to 2 cm and 6 to 8 cm from samples collected in restored and undisturbed marshes.

Sediment depth	Restoration status	pH	Salinity	NH_4_^+^	% water
0–2 cm	Restored	6.1 (0.04)*^a^*	21.1 (1.0)*^a^*	51.9 (6.6)*^a,b^*	0.65 (0.02)
	Undisturbed	5.4 (0.14)*^b^*	20.3 (1.7)*^a,b^*	99.5 (46.0)*^a,b^*	0.71 (0.02)
6–8 cm	Restored	nd	16.8 (1.4)*^b^*	61.7 (6.3)*^a^*	0.62 (0.03)
	Undisturbed	nd	19.8 (1.3)*^a,b^*	30.9 (4.8)*^b^*	0.62 (0.04)

*Different letters after the values indicate significantly different values (*P* < 0.05). No pH data were collected for sediments from 6 to 8 cm.*

### GENE ABUNDANCE PATTERNS

**FIGURE 1:**
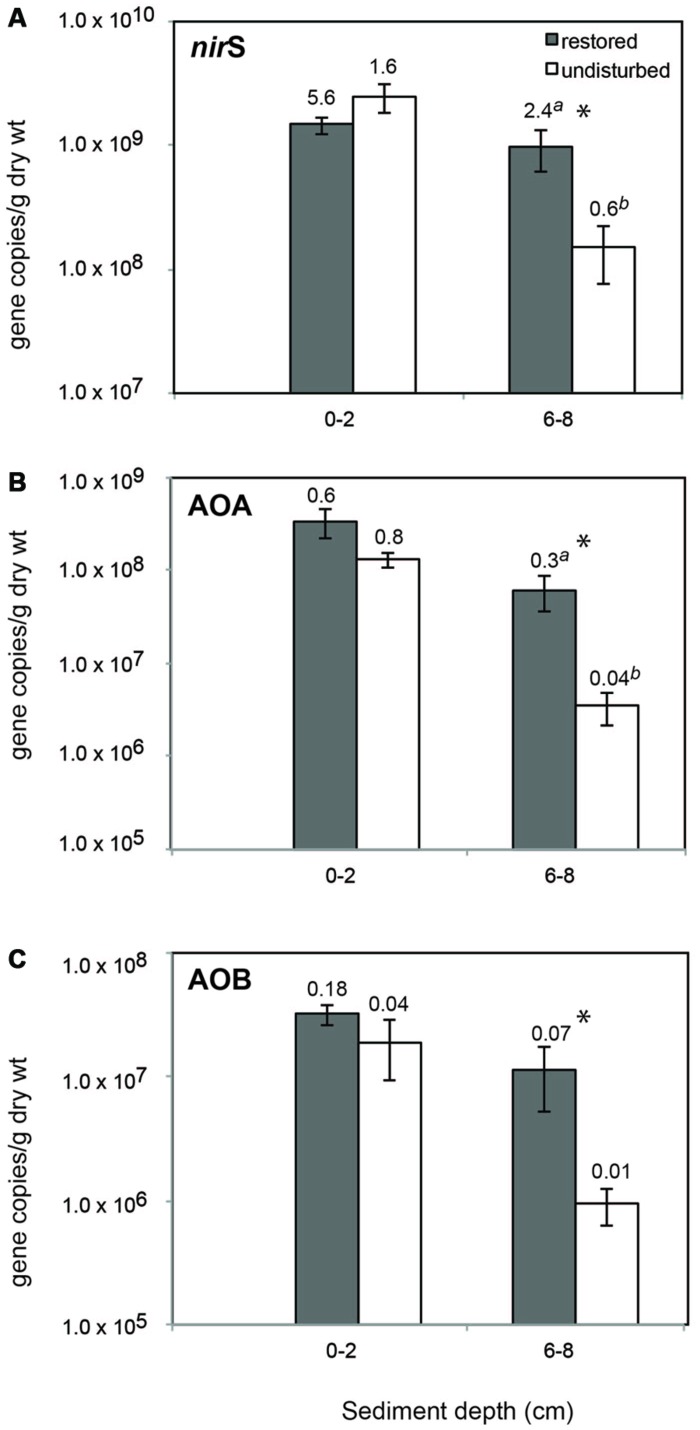
**Mean (±SE) abundance of *nir*S (A), archaeal *amo*A (B), and betaproteobacterial *amo*A (C) genes in sediment from restored and undisturbed marshes.** Asterisks (*) indicate significantly different values (*P* ≤ 0.05) between means (*n* = 4) from restored and undisturbed marshes. Numbers above bars are the mean ratio of functional gene abundance to Bacterial 16S rRNA gene abundance. Significantly different ratios between restored and undisturbed sites are indicated by different letters.

There was a consistent abundance pattern in relation to sediment depth and restoration status for all three genes with lowest abundances found in undisturbed deep sediment (**Figure 1**). For all three genes, abundance was significantly higher in restored sites compared to undisturbed sites in deep sediment only, and similar patterns were found when the data were normalized to bacterial 16S rRNA gene abundance (**Figure 1**).

Abundance of *nir*S genes ranged from 9.8 × 10^6^ to 2.1 × 10^9^ copies/gdw at the surface and 6.2 × 10^7^ to 4.6 × 10^9^ at depth, and represented as much as 9% of the total bacterial community (based on 16S rRNA gene abundance) at the surface and 36% at depth. Archaeal *amo*A genes were about one order of magnitude lower in abundance compared to *nir*S genes, and ranged from a low of 8.9 × 10^5^ in deeper sediment to 1.2 × 10^9^ at the surface. Betaproteobacterial *amo*A genes showed similar patterns, but were, on average, an order of magnitude lower than archaeal *amo*A genes, ranging from a low of 6.8 × 10^4^ (6-8 cm) to 1.2 × 10^8^ (0-2 cm). AOB and AOA comprised up to 1.6 and 4.8%, respectively, of the total bacterial community at the surface and as much as 0.6 and 1% at depth.

Abundance of *nir*S genes was positively correlated with porewater pH (Pearson’s correlation coefficient, *r* = 0.44, *P* = 0.03) and water content (*r* = 0.54, *P* = 0.007) at the restored sites, but not at the undisturbed sites. We did not detect any significant correlations between betaproteobacterial *amo*A abundance and porewater salinity, pH, ammonium, or water content.

Abundance of archaeal *amo*A genes was significantly negatively correlated with pH (*r* = -0.53, *P* = 0.0083) at the surface when restored and undisturbed sites were combined.

### COMMUNITY COMPOSITION

Ordination analysis of TRFLP profiles for denitrifiers, AOA, and AOB from marshes in southeastern Connecticut indicated different responses to disturbance among the functional groups. In all cases, over 80% of the variability was explained by the first two axes and the final stress of the ordinations suggest a low risk of drawing false inferences (McCune and Grace, 2002). Initially, we analyzed all samples combined to identify significant patterns of community composition for each gene. Using depth as the grouping variable, MRPP analysis showed significantly different communities in surface sediments compared to deeper sediments for all three genes (**Table 2**). However, we identified significant differences among communities only for *nir*S and AOB when restoration status was used as the grouping variable (both depths combined).

**Table 2:** Results from multiresponse permutation procedure (MRPP) based on TRFLP fingerprints of *nir*S, betaproteobacterial *amo*A, and archaeal *amo*A genes.

		Denitrifiying bacteria	Ammonia-oxidizing bacteria (AOB)	Ammonia-oxidizing archaea (AOA)
Depth	Grouping variable	*A*^a^	*T*^b^	*P*^c^	*A*^a^	*T*^b^	*P*^c^	*A*^a^	*T*^b^	*P*^c^
Both depths	Restoration	0.0365	-7.99	**<0.0001**	0.078	-8.78	**<0.0001**	-0.0009	0.10	0.42
	Depth	0.095	-6.88	**<0.0001**	0.065	-7.33	**<0.0001**	0.032	-3.67	**0.008**
0–2 cm only	Restoration	0.036	-0.21	**0.044**	0.007	-0.86	0.16	-0.006	0.26	0.47
6–8 cm only	Restoration	0.133	-7.96	**<0.0001**	0.208	-8.21	**<0.0001**	-0.0006	0.04	0.42

*Effects of restoration and sediment depth were tested with both depths combined. Effects of restoration only were then tested for each depth separately. Significant effects (α ≤ 0.05) are indicated by *P-*values in bold.*

*^a^*A is the intragroup average distance; when all items are identical within groups, *A* = 1.

*^b^T* = ( δ-*m*)/*s* = (observed–expected)/s. dev. of expected, where *m* and *s* are the mean and SD of δ under the null hypothesis.

*^c^P* is the probability of a smaller or equal δ.

**FIGURE 2:**
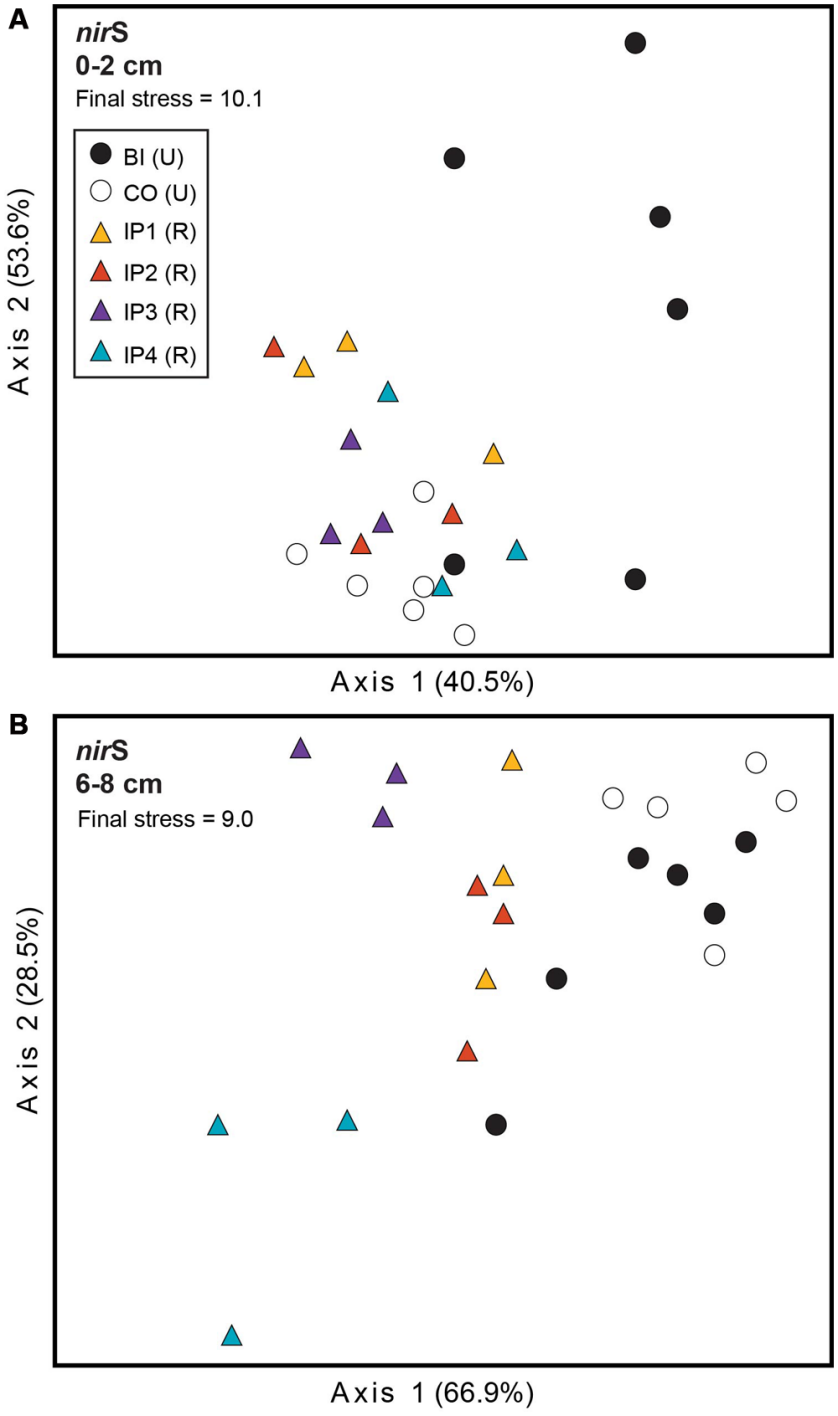
**Non-metric multidimensional scaling (NMS) plot of TRFLP profiles for *nir*S genes in surface **(A)** and deep **(B)** sediments.** Percent variability explained by each axis is shown parenthetically on the axis labels. In both panels, triangles represent restored (R) marshes (Impoundments 1–4); circles represent undisturbed (U) marshes from Barn Island (BI) or Cottrell (CO) marshes.

Further analysis of *nir*S TRFLP profiles from restored and undisturbed marshes at each depth revealed restoration effects in both sediment depths, but the effects were more striking in the deeper sediment (**Figure 2**). MRPP analysis confirmed that the denitrifier communities in restored sites were significantly different from those in undisturbed sites (**Table 2**). In surface sediments, community patterns of *nir*S genes were not distinguishable among the four impoundments, but we detected a significant site (*P* = 0.005) and marsh (*P* = 0.005) effect among the undisturbed sites from Barn Island and Cottrell marshes.

In the deeper sediments, we observed community patterns of denitrifiers indicating a chronological shift among the restored marshes from Impoundments 3 and 4 (restored in 1991 and 1987, respectively) to Impoundments 1 and 2 (restored in 1978). Impoundments 1 and 2 were more similar to each other and to undisturbed marshes, while Impoundments 3 and 4 were distinct from each other and from the other marshes (**Figure 2B**). Similar to surface sediments, undisturbed sites in Barn Island were significantly different from Cottrell marsh sites (*P* = 0.002), but undisturbed sites within each marsh were not different. When undisturbed sites from each marsh were removed from the analysis, restored sites were still significantly different from the undisturbed Barn Island marsh (*P* < 0.0001) and the Cottrell marsh (*P* < 0.0001) sites.

**FIGURE 3:**
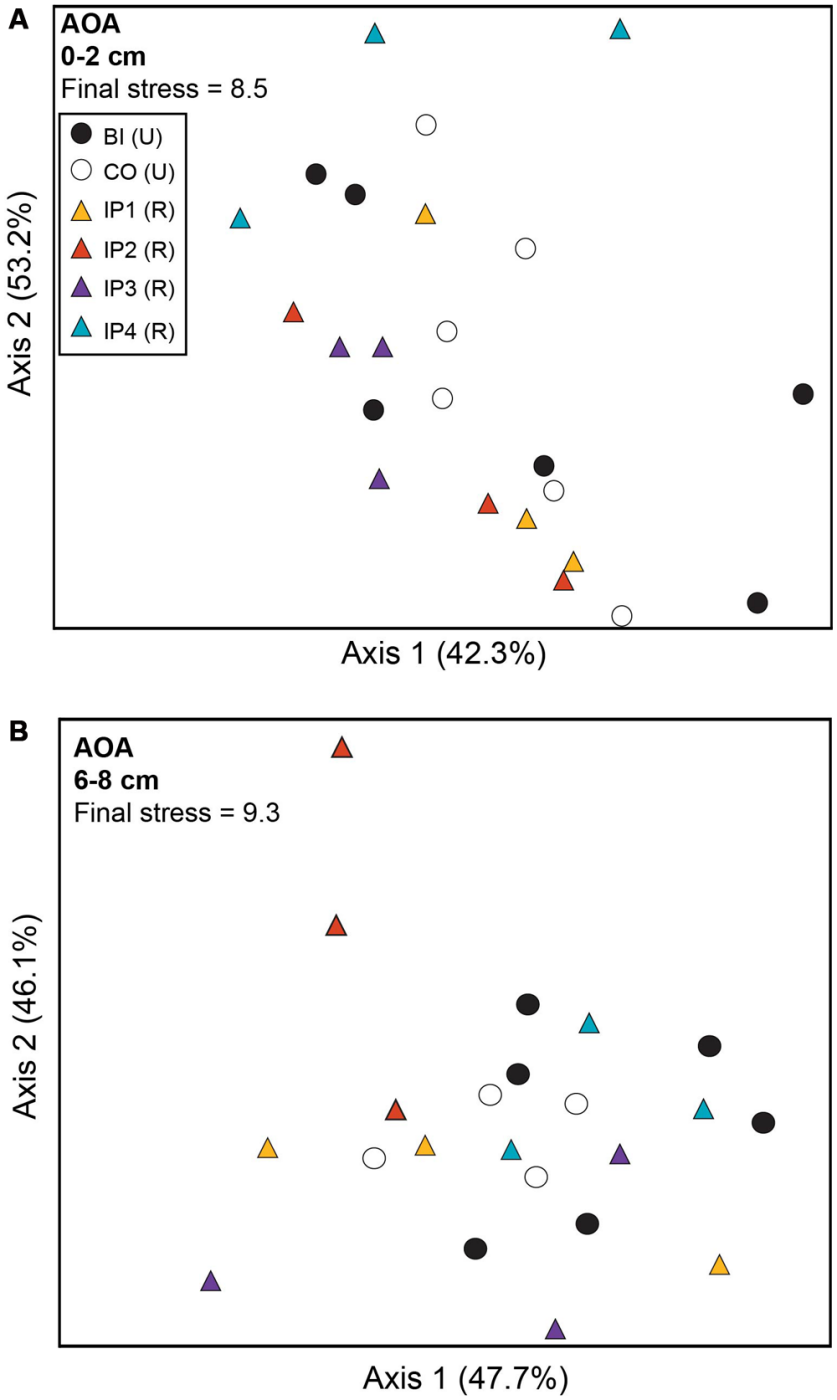
**Non-metric multidimensional scaling plot of TRFLP profiles for archaeal *amo*A genes in surface **(A)** and deep **(B)** sediments.** Percent variability explained by each axis is shown parenthetically on the axis labels. In both panels, triangles represent restored (R) marshes (IP 1–4); cirlces represent undisturbed (U) marshes from Barn Island (BI) or Cottrell (CO) marshes.

**FIGURE 4:**
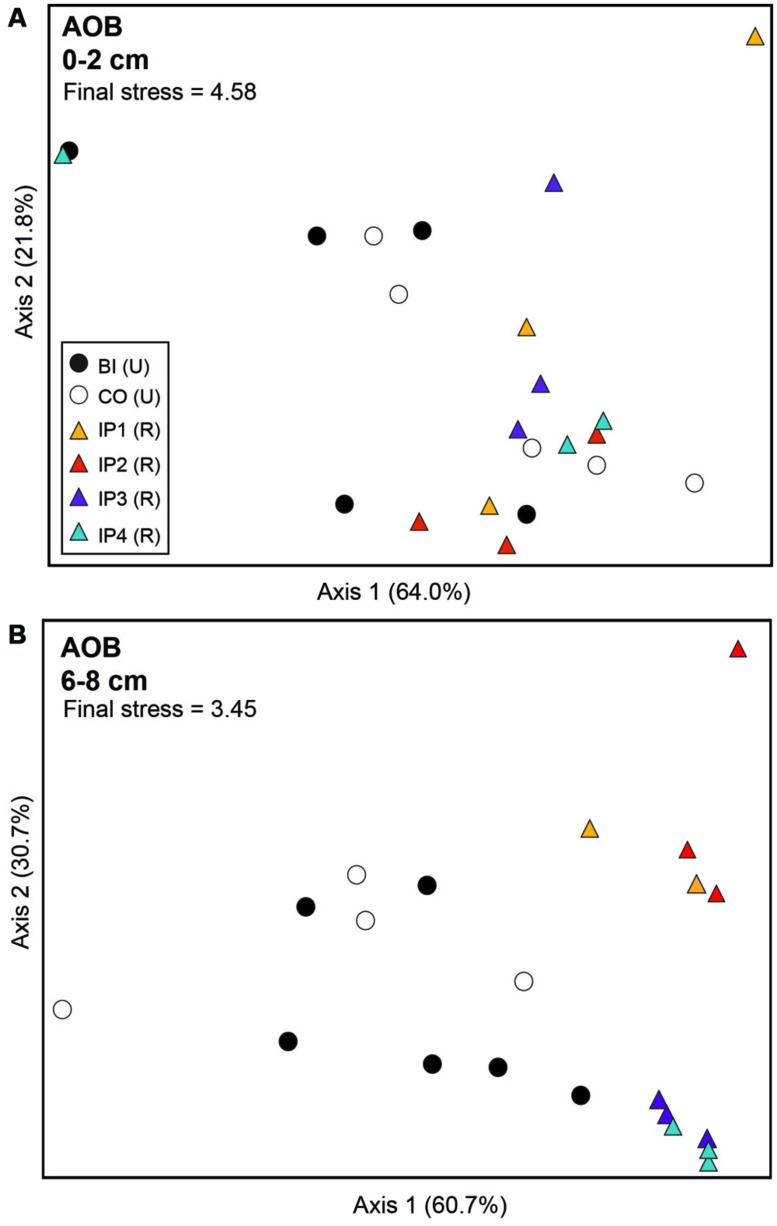
**Non-metric multidimensional scaling plot of TRFLP profiles for betaproteobacterial *amo*A genes in surface **(A)** and deep **(B)** sediments.** Percent variability explained by each axis is shown parenthetically on the axis labels. In both panels, triangles represent restored (R) marshes (IP 1–4); cirlces represent undisturbed (U) marshes from Barn Island (BI) or Cottrell (CO) marshes.

Analysis of archaeal *amo*A genes by TRFLP revealed few community differences between restored and undisturbed sites, between surface and depth, or between undisturbed Barn Island sites and Cottrell sites (**Figure 3**; **Table 2**). AOB communities, however, at restored and undisturbed marshes in surface sediments were not different, but a significant disturbance effect was detected in deeper sediment (**Figure 4**; **Table 2**). The patterns of AOB communities in deeper sediments was somewhat similar to the patterns observed for *nir*S communities, with communities at Impoundments 1 and 2 grouping together and Impoundments 3 and 4 grouping together (**Figure 4B**), but no significant differences were detected among the undisturbed sites.

Diversity of denitrifier communities based on *nir*S TRFLP profiles revealed significantly higher evenness, but not richness, in surface sediments in restored sites compared to undisturbed sites (**Table 3**). Similar patterns were also observed in the deeper sediment, but the differences were not quite significant. We detected no differences in diversity, however, of AOB or AOA communities between restored and undisturbed sites.

**Table 3:** Mean (SE) diversity indices calculated from TRF relative abundance data.

Sediment depth	Marsh status	Gene	Richness	Evenness	Shannon	Simpson
0–2 cm	Restored	*nir*S	10.9 (0.08)	**0.93 (0.004)**	**2.22 (0.010)**	**0.88 (0.001)**
	Undisturbed		10.9 (0.08)	**0.91 (0.010)**	**2.17 (0.025)**	**0.87 (0.006)**
	Restored	Archaeal *amo*A	3.9 (0.47)	0.80 (0.02)	1.02 (0.08)	0.59 (0.02)
	Undisturbed		4.8 (0.44)	0.76 (0.01)	1.14 (0.06)	0.62 (0.02)
	Restored	Bacterial *amo*A	3.90 (0.67)	0.84 (0.09)	1.10 (0.19)	0.59 (0.08)
	Undisturbed		4.00 (0.49)	0.84 (0.08)	1.18 (0.15)	0.62 (0.07)
6–8 cm	Restored	*nir*S	10.9 (0.08)	0.91 (0.01)	2.18 (0.03)	0.87 (0.01)
	Undisturbed		10.8 (0.11)	0.90 (0.01)	2.15 (0.02)	0.86 (0.01)
	Restored	Archaeal *amo*A	5.4 (0.51)	0.84 (0.03)	1.33 (0.10)	0.67 (0.03)
	Undisturbed		6.1 (0.46)	0.78 (0.01)	1.39 (0.07)	0.69 (0.02)
	Restored	Bacterial *amo*A	3.5 (0.43)	0.89 (0.02)	1.04 (0.09)	0.61 (0.03)
	Undisturbed		3.8 (0.32)	0.926 (0.01)	1.20 (0.08)	0.67 (0.03)

*Numbers in bold indicate significantly different values between restored and undisturbed marshes (*P* < 0.05).*

### PATTERNS OF TRF ABUNDANCE

**FIGURE 5:**
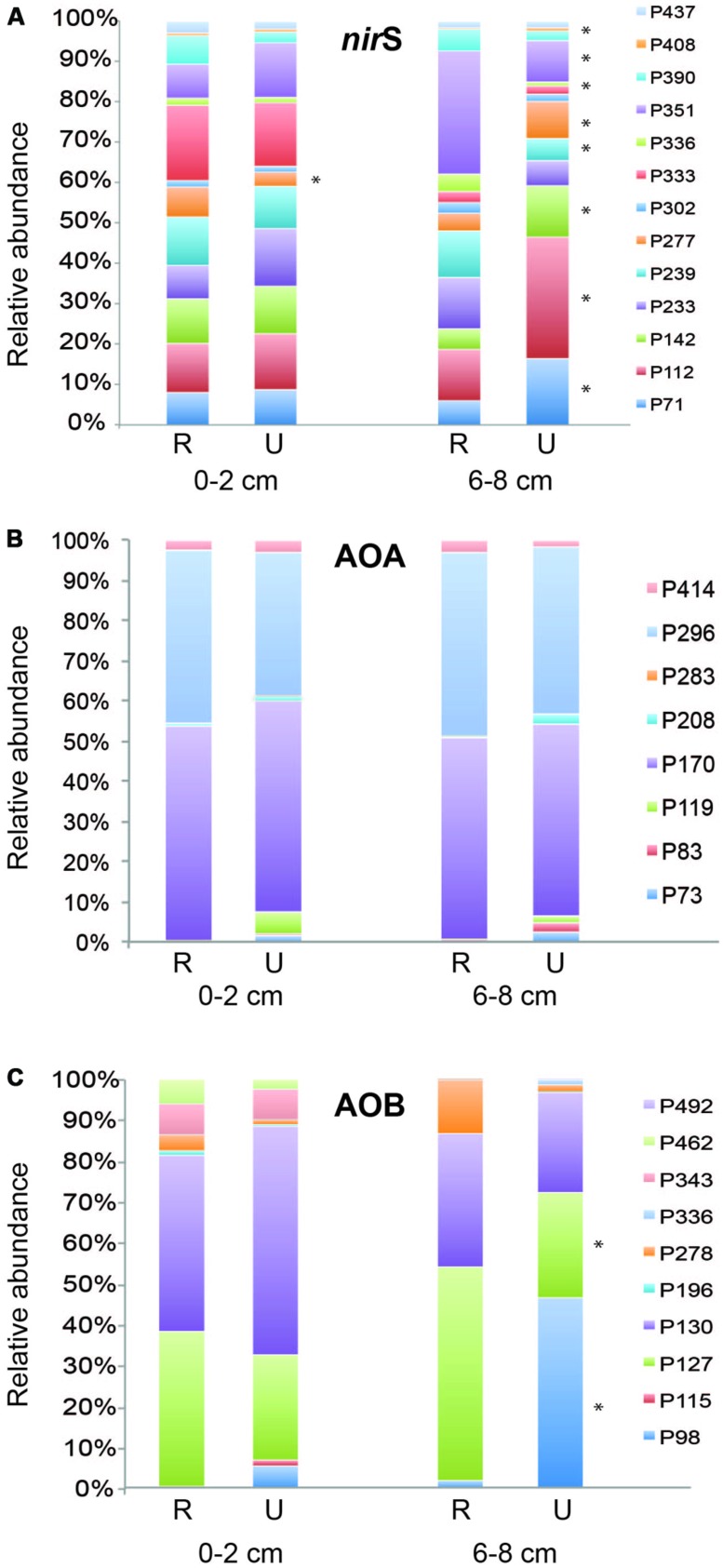
**Mean relative abundance of individual TRFs from surface and deep sediments for *nir*S **(A)**, archaeal *amo*A **(B)**, and betaproteobacterial *amo*A **(C)** genes.** Asterisks next to TRF bars indicate significantly different values (*P* ≤ 0.05) of that TRF between restored (R) and undisturbed (U) marshes.

Thirteen TRFs representing *nir*S genes were identified from analysis of over 200 *nir*S sequences from clone libraries created from both depths at one undisturbed site (Wequetequock Cove) and two restored (Impoundments 1 and 4) sites as well as from analysis of closely related published sequences (Figure S1, Table S1). Relative abundance of only one *nir*S TRF in the surface sediments was significantly different between restored and undisturbed marshes, while abundance of nine TRFs in the deeper sediments showed significant patterns related to disturbance (**Figure 5A**). Of the TRFs that showed significant differences between restored and undisturbed marshes, three of them (70, 142, and 277) showed patterns in the deeper sediments from Impoundments 1–4 that correspond to the chronology of restoration (data not shown). For example, in the deeper sediment, abundance of TRF 70 was significantly greater in undisturbed sites compared to restored sites, and was also significantly greater (*P* = 0.004) in Impoundments 1 and 2 (restored in 1978) compared to Impoundments 3 and 4 (restored 10–12 years later) when abundance from each impoundment was analyzed separately.

Archaeal *amo*A TRFs 170 and 296 were the dominant fragments detected at all sites, each comprising 40–50% of the community (**Figure 5B**). These TRFs correspond to sequences affiliated with *Nitrosopumilus* group I (TRF170) and *Nitrosopumilus* group 2 (TRF296), as reported in Peng et al. (2013). There were no significant differences in relative abundance of individual TRFs between restored and undisturbed sites. However, TRF 119 was significantly higher (*P* = 0.016) in deeper sediments compared to surface sediments at restored sites, but no differences were detected at undisturbed sites.

Betaproteobacterial *amo*A TRFs 127 and 130 were the dominant TRFs at both restored and undisturbed sites (**Figure 5C**) and have been shown in previous studies to correspond primarily to *Nitrosospira*-like *amo*A sequences (Bernhard et al., 2005; Peng et al., 2013). TRF 127 also represents a small number of sequences closely related to *Nitrosomonas* sp. NM143 (Peng et al., 2013). TRF 127 was significantly greater in relative abundance at restored sites at depth compared to undisturbed sites (*P* = 0.004). Conversely, TRF 98 was significantly greater at undisturbed sites (*P* < 0.0001), comprising nearly 50% of the community, while TRF 98 made up less than 6% of the community at other sites. TRF 98 represents sequences affiliated with the *Nitrosospira*-like cluster (Peng et al., 2013). TRFs 343 and 462 were significantly greater in surface sediments compared to deeper sediments (*P* = 0.001 and *P* = 0.003, respectively), but no differences with restoration status were detected for these two TRFs.

## DISCUSSION

In this study, we report significant impacts of chronic disturbance on coastal sediment microbial communities involved in nitrogen cycling. Our results suggest that disturbances may differentially impact different groups of microbes, and that sediment depth may be an important factor in characterizing impacts and subsequent recovery of sediment microbial communities. In a previous study of bacterial 16S rRNA genes from surface sediments from the same marshes surveyed in this study, communities were more variable at restored sites compared to undisturbed sites, but the overall community composition was similar and there were no differences in bacterial abundance (Bernhard et al., 2012). Our focus on specific functional groups, however, suggests that after nearly 30 years of restored tidal flow, there are detectable differences in abundance and community composition between restored and undisturbed marshes.

Abundance of *nir*S genes was similar to abundances reported in other salt marshes and estuaries, although in some cases our values were slightly higher than previously reported. Bowen et al. (2011) reported *nir*S abundances ranging from 10^4^ to 10^5^ copies per ng of DNA (our data converted to these units ranged from 4 × 10^4^ to 5 × 10^6^ gene copies/ng DNA) in two New England salt marshes, and ratios of *nir*S to bacterial 16S rRNA genes of 1–4%. Similarly, our *nir*S abundances were similar to those reported in San Francisco Bay estuarine sediments (Mosier and Francis, 2010), when converted to copies of *nir*S per gram wet weight. Abundances of *nir*S genes in Elkhorn Slough sediments (Smith et al., 2014), however, are at the low range of our data, with many of our samples being 1–2 orders of magnitude higher. Elkhorn Slough is an agriculturally impacted estuary, and may have different nitrogen-cycling dynamics compared to the salt marshes in Long Island Sound. The studies in San Francisco Bay and Elkhorn Slough are also from unvegetated sediments, which would also likely have different nitrogen dynamics compared to vegetated sediments in salt marshes.

Archaeal and betaproteobacterial *amo*A gene abundances at the surface are within the ranges reported in other estuaries and salt marshes (see review by Bernhard and Bollmann, 2010). Ratios of AOA and AOB to bacterial 16S rRNA genes are much lower than *nir*S ratios, which is expected for obligate chemoautotrophs. Numbers for AOA and AOB at depth have not been previously reported in these ecosystems to our knowledge. Because ammonia oxidation is an aerobic process, most studies have focused on surface sediments. However, extensive root systems in salt marshes that may provide oxygen (Mendelssohn et al., 1981; Howes et al., 1986), as well as bioturbation by invertebrates (Dollhopf et al., 2005), so it is likely that there may be aerobic micropockets that can support smaller populations of these aerobic microorganisms. The lack of a significant difference of relative AOB to bacterial 16S rRNA between restored and undisturbed marshes in deeper sediments suggests that the AOB may be less impacted compared to AOA and *nir*S, and may reflect a more general effect on the total microbial abundances rather than a specific effect on AOB.

Increased abundance of nitrifiers and denitrifiers in restored marshes compared to undisturbed marshes in deeper sediment suggests a more general impact of disturbance on nitrogen-cycling in the marsh. Increases in abundance could indicate higher rates of nitrification and denitrification at these sites. Some studies have shown strong correlations between *amo*A gene abundance and nitrification rates in estuaries for both AOA and AOB (recently reviewed in Bernhard and Bollmann, 2010). We should also note that differences in gene abundance could reflect differences in gene copy numbers per cell, rather than an increase in the population size. Some denitrifiers and AOB are known to have multiple copies of *nir*S (Etchebehere and Tiedje, 2005; Jones et al., 2008) or *amo*A (Norton et al., 1996), respectively. Multiple copies of *amo*A in AOA, however, have not been reported (Zhalnina et al., 2014).

Finding significantly greater effects of disturbance on community composition and abundance in deeper sediments relative to surface sediments suggests that the impacts of disturbance may be greater or have much longer lasting effects in deeper sediment. Impacts of disturbance may be more pronounced deeper in the sediments, since surface sediments are likely to be resuspended with each tidal cycle and redistributed across the marsh landscape, thus obscuring evidence of disturbance. Significantly different salinity between surface and deep sediments at the restored marshes, but not in the undisturbed marshes, suggests there may still be significant differences in the hydrology of the marsh. Salinity has previously been reported to be an important factor in driving nitrifier (see Bernhard and Bollmann, 2010 and references cited within) and denitrifier (Yoshie et al., 2004; Santoro et al., 2006; Bulow et al., 2008) communities in estuaries and salt marshes. Differences in hydrology would also be expected to impact oxygen levels. When marshes are tidally restricted, the water table drops, allowing increased oxygen penetration, and the sediment subsides (Portnoy, 1999). Once seawater is restored, there may be significant differences in porewater chemistry due to the lower elevation.

Additionally, Swamy et al. (2002) reported significantly different densities of some benthic invertebrates in restored marshes in Barn Island compared to reference marshes, which may significantly impact sediment turnover and C and N distributions (Wang et al., 2010), as well as oxygenation of deeper sediments. Furthermore, since root and rhizome densities may vary with sediment depth (e.g., Bertness, 1985), there may also be depth-specific differences in the relative importance of plant activity on how microbes grow and recover after disturbance. Previous studies in soils and sediments have suggested a significant effect of plant root exudates on denitrifying activity (Kaplan et al., 1979; Christensen and Sørensen, 1986; Risgaard-Petersen and Jensen, 1997; Henry et al., 2008). Root exudates are also known to significantly impact oxygen in sediments (Mendelssohn et al., 1981; Howes et al., 1986).

Significantly different NH_4_^+^ concentrations between restored and undisturbed marshes in deeper sediments suggests differences in resource availability for nitrifiers. If nitrification and denitrification are coupled (Risgaard-Petersen, 2003), nitrifier resource availability would be expected to impact denitrifiers as well. Higher ammonium in restored sites compared to undisturbed sites may reflect differences in mineralization of N due to increased decomposition. Others have reported increased decomposition via sulfate reduction in impounded and restored marshes compared to reference marshes (van Proosdij et al., 2010). Portnoy and Giblin (1997) also reported increased N and P mineralization and increased sulfides, suggesting accelerated rates of sulfate reduction in restored marshes.

The recovery trajectory implied by the community patterns for *nir*S and betaproteobacterial *amo*A genes support a repeatable pattern for recovery of these genes in deep sediment. The lack of a similar pattern in surface sediments may indicate that only certain communities show reproducible recovery patterns or that certain edaphic conditions are more likely to lead to these patterns. Bach et al. (2010) reported a directional shift in microbial communities in silty clay soils, but not in sandy loam soils. Similarly, Levine et al. (2011) reported a recovery trajectory for methanotrophs, but not for heterotrophic bacteria, while Banning et al. (2011) reported broad support for successional changes at the phylum level in soil bacterial communities. These seemingly contrary results suggest that either sampling or methodology was inadequate to detect the patterns, or that recovery or succession of some communities is not as predictable or reproducible as others. Recently, Pagaling et al. (2014) demonstrated that historical contingencies also play a role in determining predictable patterns of succession.

The directional shift in *nir*S communities may provide some insight into how long it takes for communities to recover, or at least become indistinguishable from communities in undisturbed marshes. Tidal inundation was restored to Impoundments 1 and 2 in 1978, and the denitrifying communities at these sites are more similar to those in undisturbed marshes compared to the denitrifying communities in Impoundments 3 and 4, which did not have tidal flow restored until 10–12 years later. Others have reported similarly long-lasting effects of disturbance on soil microbial communities (Bach et al., 2010; Levine et al., 2011) and have found strong correlations of microbial community recovery with soil texture (Bach et al., 2010), edaphic conditions such as pH, C, N, and P availability (Banning et al., 2011), and microbial immigration rates (Lambert et al., 2012).

We also identified specific denitrifier populations (i.e., specific TRFs) that were significantly different between restored and undisturbed sediment. Unfortunately, in almost all cases, TRFs represent polyphyletic groups, so we cannot relate specific phylogenetic clusters with recovery. However, several TRFs show patterns that are consistent with a recovery trajectory. TRFs 71, 142, and 277 are significantly more abundant in the undisturbed marshes, and also show a statistically significant increase from their abundance in Impoundments 3 and 4 to Impoundments 1 and 2, as you would predict if these denitrifiers are representative of undisturbed or recovered marshes. Following changes in these TRFs may provide a monitoring mechanism for the recovery of denitrifying communities in salt marshes. Future sampling in the Barn Island marshes will determine whether the recovery trajectory of denitrifiers is on track to return to resemble those of undisturbed marshes.

Significant site effects among the undisturbed sites for *nir*S genes in the surface sediments suggest that restoration to pre-disturbance conditions may be impossible to fully assess given the variation among undisturbed sites. However, since there was no site effect among the restored sites and all of the undisturbed sites were significantly different from the restored sites, the data still support a significant disturbance effect in the marsh.

Although the patterns for AOB do not indicate an obvious recovery trajectory among the four impoundments as for denitrifiers, the relative abundance patterns for TRFs 98 and 127 in deeper sediment suggest differential responses to disturbance. Additional experiments, however, are necessary to explore this hypothesis.

Disturbance effects were also detected in diversity patterns for *nir*S genes. However, we interpret the data with caution since *nir*S TRFs show little correlation to phylogeny and each TRF represents multiple sequence types and sequence types are represented by multiple TRFs. However, Shannon indices were just slightly lower than those previously reported for estuarine sediments based on *nir*S sequences rather than TRF patterns (Santoro et al., 2006). Other studies have shown significant effects of disturbance on microbial diversity (Wittebolle et al., 2009; Levine et al., 2011). More sequences are necessary to fully describe *nir*S diversity.

Recent studies of disturbance on salt marsh microbial communities have revealed surprisingly few effects (Bowen et al., 2009, 2011; Bernhard et al., 2012; Peng et al., 2013), even though the studies were conducted in different marshes with different disturbance regimes, used different methods, and focused on different microbial groups. It is possible that because the studies were done many years after the initial disturbances, any alterations in the microbial communities may have been missed. However, in some cases, macrobiota and biogeochemical processes were significantly different following disturbance and have remained different many years post-disturbance, yet analysis of the microbial communities from the same areas suggest a surprising degree of resilience or resistance to perturbations. It is also possible that the microbes present in these marshes show a surprising degree of phenotypic plasticity as the conditions change, thus leading to changes in process rates, but no detectable change in the community composition.

An alternative hypothesis, however, is that changes may vary with sediment depth. One commonality of the studies is that they focused only on surface (the top 2 cm or less) sediments, yet we found greater impacts of disturbance in deeper (6–8 cm) sediments. In a previous study of the same sites as presented here, Bernhard et al. (2012) reported no differences in abundance of bacterial 16S rRNA genes in surface sediments. Based on the depth-specific patterns we observed for functional genes in the current work, we decided to measure bacterial 16S rRNA gene abundance from the same deep sediments. In support of our hypothesis, we found significantly higher abundance of bacterial 16S rRNA genes in the 6–8 cm sediments from restored sites (Bernhard, unpublished), thus echoing the patterns of abundance for *nir*S in restored and undisturbed sites. Additionally, in a study of recovery in a freshwater wetland (Meyer et al., 2008), most of the changes in N and C pools occurred within the surface across a 10-year chronosequence.

It will be interesting to conduct additional community composition studies based on bacterial 16S rRNA to determine if the bacterial communities may also differ significantly at depth. Although the data are limited, we believe further investigation is warranted and may change the conclusions from previous studies of disturbance on salt marsh microbial communities if more “in-depth” analyses are conducted in the future. We must also report that recently, Bowen et al. (2013) was able to detect small differences in the gene sequences of denitrifying communities in surface sediments (0–1 cm) in fertilized plots compared to unfertilized plots after analyzing more than 60,000 *nir*S sequences, suggesting that we may just need to look harder to identify differences in surface sediments.

In conclusion, our study suggests that salt marsh nitrogen-cycling microbial communities may be impacted by disturbance, but it is uncertain whether the altered communities are functionally similar to those in undisturbed marshes. If there is a link between community composition, gene abundance, and process rates, as some studies have documented for nitrifiers and denitrifiers (e.g., Cavigelli and Robertson, 2000; Webster et al., 2005; Bernhard et al., 2007; Philippot et al., 2013), then our data point strongly toward differences in nitrogen-cycling in disturbed areas of the marsh compared to undisturbed marshes. However, in a study of disturbance on soil denitrifiers, Wertz et al. (2007) found that a community may recover functionally even though the diversity remains altered, suggesting some level of functional redundancy. Similarly, Cao et al. (2008) reported that denitrifier community composition and function were uncoupled in a California salt marsh. Whether the communities of nitrifiers and denitrifiers function similarly in disturbed and undisturbed marshes or not, it is important to understand how microbial communities respond to disturbance and to identify underlying principles that govern the changes.

## AUTHOR CONTRIBUTIONS

Anne E. Bernhard designed the project, provided QPCR data, and wrote the manuscript; Courtney Dwyer and Adrian Idrizi provided community fingerprints, analyses, and interpretation; Rachel Zwick and Geoffrey Bender provided sequence data, phylogenetic analyses, and interpretations, and Geoffrey Bender helped with sample collection and processing. All authors contributed to the intellectual content and editing of drafts.

## Conflict of Interest Statement

The authors declare that the research was conducted in the absence of any commercial or financial relationships that could be construed as a potential conflict of interest.
