# Supplementary material for: Long-term impacts of disturbance on nitrogen-cycling bacteria in a New England salt marsh
Source: Front Microbiol. 2015 Feb 4;6:46. doi: 10.3389/fmicb.2015.00046 (PMC4316780; doi:10.3389/fmicb.2015.00046)
Supplement: Supplementary file 1 [file Data_Sheet_1.PDF]

Supplemental data provides information about the distribution of operational taxonomic units and TRFs (Table S1), and phylogenetic relationships of *nir*S sequences used to identify terminal restriction fragments (Figure S1).

Table S1. Operational Taxonomic Units (OTUs) for *nir*S sequences representing each Terminal Restriction Fragment (TRFs) identified by sequence analysis from *nir*S gene clone libraries generated from one undisturbed site (WE) and two restored sites (Impoundments 1 and 4). OTUS were identified as ≥ 95% nucleotide identity using MOTHUR (Schloss et al. 2009). OTUs that are underlined indicate those that are represented by more than one TRF. Sequences representing each OTU are shown in Figure S1.

TRF	OTU
**36**	1, 9, 10, 26, 27, 32
**70**	3, 11, 12, 13, 15, 19, 24, 43, 55, 57, 60, 63, 64
**112**	5, 6, 8, 14, 17, 22, 28, 30, 31, 35, 44, 45, 51, 53
**142**	3, 46
**234**	4, 7, 21
**239**	14, 20, 47, 50, 62
**277**	8, 18, 38, 59
**302**	61, 65
**335**	48
**337**	1, 37, 40, 52
**390**	41
**408**	58
**437**	2, 12, 42, 54

Figure S1. Phylogenetic relationships among deduced amino acid sequences for the dissimilatory nitrite reductase gene (*nir*S). The neighbor-joining tree was inferred from 252 amino acid residues generated from cloned *nir*S amplicons using primers *nir*S-1F and *nir*S-6R (Braker et al. 1998). Bootstrap values greater than 50 (computed from 100 replicates) are shown. Bootstrap values greater than 50 and 80 computed from parsimony analysis that support the neighbor-joining topology (100 replicates) are indicated by gray and black circles, respectively. *Hydrogenobacter thermophilus nir*S (AB210046) was used as the outgroup. Only near full-length sequences were included, so not all OTUs are represented in the tree. Nucleotide sequences for *nir*S have been deposited in Genbank under the accession numbers KF895915-KF896071.

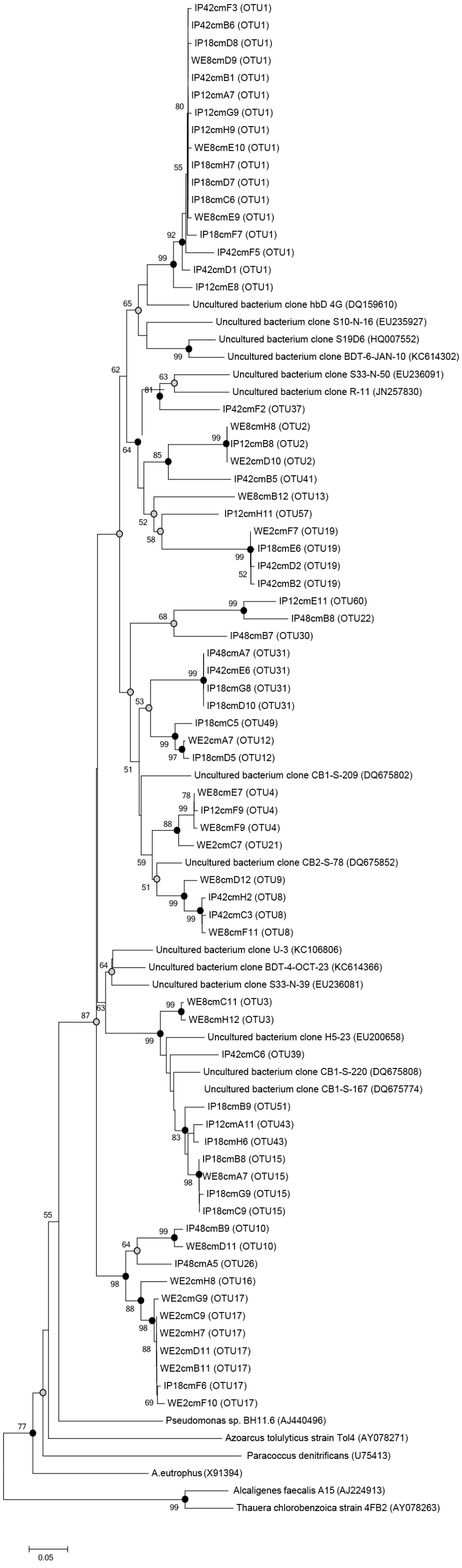
IP42cmF3 (OTU1)
IP42cmB6 (OTU1)
IP18cmD8 (OTU1)
WE8cmD9 (OTU1)
IP42cmB1 (OTU1)
IP12cmA7 (OTU1)
IP12cmG9 (OTU1)
IP12cmH9 (OTU1)
WE8cmE10 (OTU1)
IP18cmH7 (OTU1)
IP18cmD7 (OTU1)
IP18cmC6 (OTU1)
WE8cmE9 (OTU1)
IP18cmF7 (OTU1)
IP42cmF5 (OTU1)
IP42cmD1 (OTU1)
IP12cmE8 (OTU1)
Uncultured bacterium clone hbD 4G (DQ159610)
Uncultured bacterium clone S10-N-16 (EU235927)
Uncultured bacterium clone S19D6 (HQ007552)
Uncultured bacterium clone BDT-6-JAN-10 (KC614302)
Uncultured bacterium clone S33-N-50 (EU236091)
Uncultured bacterium clone R-11 (JN257830)
IP42cmF2 (OTU37)
WE8cmH8 (OTU2)
IP12cmB8 (OTU2)
WE2cmD10 (OTU2)
IP42cmB5 (OTU41)
WE8cmB12 (OTU13)
IP12cmH11 (OTU57)
WE2cmF7 (OTU19)
IP18cmE6 (OTU19)
IP42cmD2 (OTU19)
IP42cmB2 (OTU19)
IP12cmE11 (OTU60)
IP48cmB8 (OTU22)
IP48cmB7 (OTU30)
IP48cmA7 (OTU31)
IP42cmE6 (OTU31)
IP18cmG8 (OTU31)
IP18cmD10 (OTU31)
IP18cmC5 (OTU49)
WE2cmA7 (OTU12)
IP18cmD5 (OTU12)
Uncultured bacterium clone CB1-S-209 (DQ675802)
WE8cmE7 (OTU4)
IP12cmF9 (OTU4)
WE8cmF9 (OTU4)
WE2cmC7 (OTU21)
Uncultured bacterium clone CB2-S-78 (DQ675852)
WE8cmD12 (OTU9)
IP42cmH2 (OTU8)
IP42cmC3 (OTU8)
WE8cmF11 (OTU8)
Uncultured bacterium clone U-3 (KC106806)
Uncultured bacterium clone BDT-4-OCT-23 (KC614366)
Uncultured bacterium clone S33-N-39 (EU236081)
WE8cmC11 (OTU3)
WE8cmH12 (OTU3)
Uncultured bacterium clone H5-23 (EU200658)
IP42cmC6 (OTU39)
Uncultured bacterium clone CB1-S-220 (DQ675808)
Uncultured bacterium clone CB1-S-167 (DQ675774)
IP18cmB9 (OTU51)
IP12cmA11 (OTU43)
IP18cmH6 (OTU43)
IP18cmB8 (OTU15)
WE8cmA7 (OTU15)
IP18cmG9 (OTU15)
IP18cmC9 (OTU15)
IP48cmB9 (OTU10)
WE8cmD11 (OTU10)
IP48cmA5 (OTU26)
WE2cmH8 (OTU16)
WE2cmG9 (OTU17)
WE2cmC9 (OTU17)
WE2cmH7 (OTU17)
WE2cmD11 (OTU17)
WE2cmB11 (OTU17)
IP18cmF6 (OTU17)
WE2cmF10 (OTU17)
Pseudomonas sp. BH11.6 (AJ440496)
Azoarcus tolulyticus strain Tol4 (AY078271)
Paracoccus denitrificans (U75413)
A.eutrophus (X91394)
Alcaligenes faecalis A15 (AJ224913)
Thauera chlorobenzoica strain 4FB2 (AY078263)
0.05

References

Braker, G., A. Fesefeldt and K. P. Witzel. (1998). Development of PCR primer systems for amplification of nitrite reductase genes (nirK and nirS) to detect denitrifying bacteria in environmental samples. *Appl. Environ. Microbiol.* **64**: 3769-3775.

Schloss, P. D., S. L. Westcott, T. Ryabin, J. R. Hall, M. Hartmann, E. B. Hollister, R. A. Lesniewski, B. B. Oakley, D. H. Parks, C. J. Robinson, J. W. Sahl, B. Stres, G. G. Thallinger, D. J. Van Horn and C. F. Weber. (2009). Introducing mothur: Open-source, platform-independent, community-supported software for describing and comparing microbial communities. *Appl Environ Microbiol* **75**: 7537-7541.
